# *In Vitro* and *In Vivo* Models of Cerebral Ischemia Show Discrepancy in Therapeutic Effects of M2 Macrophages

**DOI:** 10.1371/journal.pone.0067063

**Published:** 2013-06-25

**Authors:** Virginie Desestret, Adrien Riou, Fabien Chauveau, Tae-Hee Cho, Emilie Devillard, Marilena Marinescu, René Ferrera, Catherine Rey, Marie Chanal, Denis Angoulvant, Jérôme Honnorat, Norbert Nighoghossian, Yves Berthezène, Serge Nataf, Marlène Wiart

**Affiliations:** 1 CNRS, UMR 5220; INSERM, U1044; INSA de Lyon; Centre de recherche en acquisition et traitement de l’image pour la santé (CREATIS), Lyon, France; 2 Hospices Civils de Lyon (Lyon University Hospitals), Lyon, France; 3 Université de Lyon, Lyon, France; 4 INSERM, U1028; CNRS, UMR 5292; Centre de recherche en neurosciences de Lyon (CRNL), Lyon, France; 5 INSERM, U886, Lyon, France; 6 ProfilXpert, UNIV-US7 INSERM; UMS 3453 CNRS, Lyon, France; Julius-Maximilians-Universität Würzburg, Germany

## Abstract

The inflammatory response following ischemic stroke is dominated by innate immune cells: resident microglia and blood-derived macrophages. The ambivalent role of these cells in stroke outcome might be explained in part by the acquisition of distinct functional phenotypes: classically (M1) and alternatively activated (M2) macrophages. To shed light on the crosstalk between hypoxic neurons and macrophages, an *in vitro* model was set up in which bone marrow-derived macrophages were co-cultured with hippocampal slices subjected to oxygen and glucose deprivation. The results showed that macrophages provided potent protection against neuron cell loss through a paracrine mechanism, and that they expressed M2-type alternative polarization. These findings raised the possibility of using bone marrow-derived M2 macrophages in cellular therapy for stroke. Therefore, 2 million M2 macrophages (or vehicle) were intravenously administered during the subacute stage of ischemia (D4) in a model of transient middle cerebral artery occlusion. Functional neuroscores and magnetic resonance imaging endpoints (infarct volumes, blood-brain barrier integrity, phagocytic activity assessed by iron oxide uptake) were longitudinally monitored for 2 weeks. This cell-based treatment did not significantly improve any outcome measure compared with vehicle, suggesting that this strategy is not relevant to stroke therapy.

## Introduction

It is now acknowledged that inflammation plays a major pathophysiological role in ischemic stroke [1]. The inflammatory response is dominated by innate immune cells of myeloid lineage: resident microglia and blood-derived macrophages. However, it remains to be shown whether the impact of macrophages on the outcome of brain ischemia is deleterious [2] or beneficial [3]. As they penetrate tissue, they respond to microenvironmental signals, acquiring distinct functional phenotypes: classically (M1) or alternatively activated (M2) macrophages. This versatile plasticity may at least partly explain their ambivalent impact under pathological conditions. M2 macrophages are important in resolving the inflammatory response and play a part in debris scavenging, tissue remodeling, and angiogenesis [4]. They were shown to support neurorepair in various models of central nervous system lesion [5], [6], [7]. In permanent focal cerebral ischemia, protective mechanisms induced by M2 activation have been shown to come into play soon after injury (24–48 h) and to last at least 7 days when neuron phagocytosis and debris removal are prevalent [8]. In transient focal ischemia, M2-type genes were found to be induced as of 1 to 3 days after injury, peaking at 3 to 5 days. The majority of M2-type genes began to decrease at 7 days and returned to pre-injury levels by day 14 [9].

Elucidating the impact of blood-derived macrophages on neuron viability after focal cerebral ischemia is of great interest, as it may lead to new therapeutic strategies for stroke patients. This is especially important given the current lack of treatment options apart from thrombolysis, which benefits only a very small proportion of patients. The first study objective was therefore to investigate the influence of blood-derived macrophages on neuron survival under ischemic conditions. The interplay between the 2 cell types was modeled by co-culturing bone marrow-derived macrophages with organotypic hippocampal slice cultures (OHCs) that had been subjected to oxygen-glucose deprivation (OGD). The results showed that macrophages provided potent protection against neuron cell loss, through a paracrine mechanism. What is more, they expressed M2-type alternative polarization. These findings raised the possibility of using bone marrow-derived M2 macrophages as a cell-based intervention (CBI) for stroke. Consequently, the second objective was to assess the effect of M2 macrophage administration in an *in vivo* model of transient focal cerebral ischemia.

The lack of translational success observed in stroke neuroprotection trials conducted thus far has led the community to propose frameworks intended to improve the quality of preclinical stroke studies [10], in particular in the field of CBI [11]. *In vivo* experiments were therefore conducted in compliance with these guidelines, with non-invasive MRI follow-up.

## Materials and Methods

### Animals

Animal investigations conformed to the Guide for the Care and Use of Laboratory Animals published by the European Union (EEC Council Directive 86/609). Protocol approval was granted by our University ethics review board (CeLyne: *Comité d’Ethique Lyonnais pour les Neurosciences Expérimentales*).

### Overall Protocols

#### In vitro study

OHCs were prepared as detailed hereafter and cultured for 2 days before being subjected to OGD for 30 min and then further cultured for 2 days under normoxic normoglycemic conditions, with or without bone marrow-derived macrophage co-culture; they were then examined by immunofluorescence.

#### In vivo study

**Figure 1:**
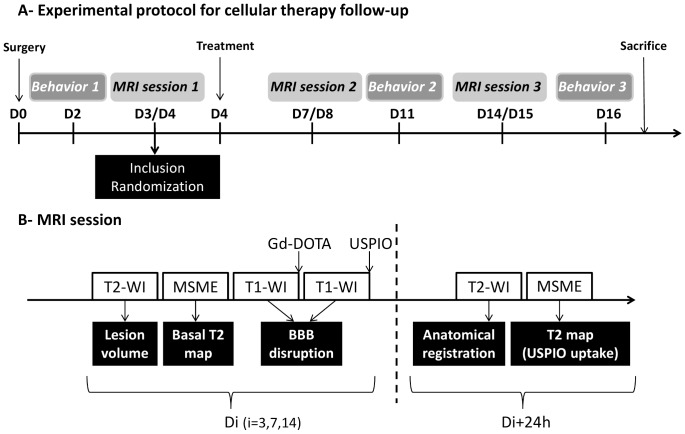
Experimental protocol and diagram of MRI session. (**A**) Experimental protocol. Surgery consisted in either transient middle cerebral artery occlusion (tMCAO) or sham operation. Treatment consisted in intravenous injection of either 2 million M2 macrophages diluted in 1 ml phosphate-buffered saline (PBS) or the same volume of pure PBS. The number of animals included is given in Table 1. (**B**) Diagram of one MRI session. T1/T2-WI: T1/T2-weighted imaging; MSME: Multi-Slice Multi-Echo; USPIO: ultrasmall superparamagnetic particles of iron oxide. BBB: blood-brain barrier. The sequence parameters are given in Table 3.

The experimental design is shown in Figure 1A, and the number of animals in Table 1. In brief, surgery (transient middle cerebral artery occlusion (tMCAO) or sham) was performed on day 0 (D0). All animals were then imaged by MRI on D3. Exclusion criteria were based on this first MRI session: 1) signs of subarachnoid or intracerebral hemorrhage, and 2) an ischemic lesion confined to the striatum without cortical involvement. Animals included at D3 were randomly assigned to one of two treatment groups: 2 million M2 macrophages or control (vehicle). Treatments were administered intravenously (1****ml over 5 minutes) at D4. All included animals were followed up longitudinally for 2 weeks post-surgery. Therapeutic benefit was evaluated before (baseline) and after treatment by sensorimotor testing and monitoring the following MRI endpoints: lesion size and brain swelling (T2-weighted imaging), blood-brain barrier (BBB) integrity (T1-weighted imaging before and after injection of DOTA-gadolinium) and phagocytic activity as neuroinflammation marker (quantitative T2 values before and 24 h after injection of ultrasmall particles of iron oxide (USPIO)). USPIO injections were randomized so as also to constitute a control non-USPIO group. Animals were sacrificed at D16 after the last behavioral examination, and prepared for immunohistological analysis. All examiners assessing MRI, behavior and histopathological data were blinded to treatment allocation.

**Table 1:** Number of animals included.

Rats operated: 31	tMCAO: 24	tMCAO rats excluded based on D3 MRI: 8			Included in analysis*
		tMCAO rats included:16	M2-treated: 8	with USPIO: 5	8
				without USPIO: 3	
			PBS-treated: 8	with USPIO: 5	8
				without USPIO: 3	
	Sham: 7	Sham rats excluded based on D3 MRI: 0			Included in analysis*
		Sham rats included: 7	M2-treated: 2	with USPIO: 2	2
			PBS-treated: 5	with USPIO: 5	3 (2 animals excluded because of incomplete data sets)

* Rats that had a complete set of longitudinal MRI data.

tMCAO: transient middle cerebral artery occlusion; PBS: Phosphate buffer saline; USPIO: Ultrasmall superparamagnetic particles of iron oxide.

### Organotypic Hippocampal Culture

Hippocampal slices were prepared from 7 day-old C57Bl6J mice (Charles River, France) as previously described [12]. Briefly, hippocampi were rapidly removed under sterile conditions, placed into ice-cold medium and cut into transverse 200-µm slices using a McIllwain tissue chopper. Slices were then cultured on semi-permeable membrane inserts (Millipore, Bedford, MA, USA), in 6-well culture plates containing 1 ml culture medium per well (composition: 50% Dulbecco’s Modified Eagle Medium (DMEM) (Invitrogen, Carlsbad, CA, USA), 25% normal horse serum, 25% Hank’s Buffer Salt Solution, 1% L-glutamine, 1% penicillin-streptomycin and 6.5 mg/ml glucose. OHCs were cultured for 2 days at 37°C in a 5% CO_2_-enriched atmosphere before OGD.

### Oxygen-glucose Deprivation

Slices were washed 3 times in serum- and glucose-deprived medium (75% basal medium essential, 25% Hank’s Buffer Salt Solution, 1% L-glutamine) before being transferred to a hypoxic chamber (95% N_2_, 5% CO_2_). Hypoxia was performed by incubation in an air-tight humidified glass chamber (Verretech, Lyon, France) at 37°C ventilated with N_2_ 95%+CO_2_ 5%. OHCs were subjected to OGD for 30 min at 37°C and then further cultured for 2 days in serum-free glucose-replete medium (75% basal medium essential, 25% Hank’s Buffer Salt Solution, 2% B27, 1% L-glutamin and 6.5 mg/ml glucose) under normoxic conditions, as previously described [12]. As appropriate, OHCs were co-cultured for 2 days with bone marrow-derived macrophages after OGD. Bone marrow-derived macrophages were cultured on the plastic surface of 6-well plates, to prevent any physical contact with the hippocampal slices cultured on permeable membrane inserts. Three independent experiments were performed.

### Primary Culture of Bone Marrow-derived Macrophages

Animals were sacrificed by intraperitoneal injection of pentobarbital, and bone marrow cells were harvested by flushing out tibias and femurs. Total bone marrow cells were then seeded on uncoated 6-well plates at 5×10^5^ cells/ml in Iscove’s Modified Dulbecco’s Medium (IMDM) (Invitrogen, Carlsbad, CA, USA) supplemented with 15% fetal calf serum (Fetal Clone II, Hyclone), penicillin-streptomycin (1 µg/ml) and 10 ng/ml human macrophage-colony stimulating factor (hM-CSF) (PrepoTech Inc, Rocky Hill, NJ, USA). Cultures were incubated at 37°C in a moist 5% CO_2_-95% air. Seven days after plating, non-adherent cells were harvested and adherent cells were washed twice in PBS.

As appropriate, bone marrow-derived macrophages were stimulated with murine Interferon-γ (50 ng/ml) (PrepoTech Inc, Rocky Hill, NJ, USA) for 18 h or murine Interleukin-4 (50 ng/ml) (PrepoTech Inc, Rocky Hill, NJ, USA) for 48 h, to generate M1 classically-activated macrophages or M2 alternatively-activated macrophages respectively, as previously described [13], [14]. Otherwise, bone marrow-derived macrophages were not further stimulated (M0 macrophages). Macrophage cell culture purity under these experimental conditions was repeatedly found to exceed 95% on immunocytofluorescence or FACS analysis of CD11b+ cells (data not shown).

### Immunohistofluorescence

Hippocampal slices were fixed with 4% paraformaldehyde for 10 min at room temperature. They were then rinsed 3 times in PBS (Euromedex, Mundolsheim, France) and incubated for 30 min at room temperature with a blocking solution of 4% bovine serum albumin (IDbio, Limoges, France) diluted in PBS and supplemented with 10% normal goat serum (Vector laboratories, Burlingame, CA, USA). They were then incubated overnight at 4°C with mouse anti-mouse neuronal nuclei (NeuN) antibody (Chemicon International, Temecula, CA, USA). Antibodies were diluted 1∶100 in blocking solution. Tissues were then rinsed 3 times in PBS and incubated with a biotinylated goat anti-mouse (Molecular Probes, Saint-Aubin, France) for 1 h at room temperature. After 3 washes, they were finally incubated with streptavidin-Alexafluor (488 or 506, Molecular probes, Saint-Aubin, France) diluted 1∶100 in PBS. 4′, 6-Diamidine-2-phenylindole dihydrochloride (DAPI, Roche, Mannheim, Germany) nuclear staining was performed and the slides were mounted using Fluoroprep (BioMerieux, Marcy l’Etoile, France).

### Immunohistofluorescence Image Analysis

To assess neuron cell loss in the hippocampal slices, the surface covered by NeuN staining was measured in the dentate gyrus. Images from selected areas were digitally photographed under high magnification using a fluorescent microscope (Zeiss Axioplan II) coupled to a CDD camera (F-View II; Soft Imaging System). Image analysis was then performed using analySIS software (analySIS 3.0; Soft Imaging System). A 0.04 mm^2^ region of interest (ROI) was first defined and measured in the selected area. Then, the area occupied by NeuN staining was determined by automatic measurement of the area of pixels above a threshold value. Finally, the NeuN immunostaining area was indexed to the total ROI area, thereby estimating the density of NeuN staining. In each experiment (n = 3), measures obtained in control OHCs were attributed the arbitrary value of 100%, with all other measures expressed as a percentage of this value.

### mRNA Extraction and Reverse Transcription

mRNAs were extracted with the RNeasy Mini Kit (Qiagen, Courtaboeuf, France) from macrophages that had been co-cultured with OHCs, M0 macrophages (non-stimulated), M1 macrophages and M2 macrophages. Total RNA (20 ng) was then reverse transcribed and cDNA amplified using the QuantiTect Whole Transcriptome kit (Qiagen, Courtaboeuf, France).

### Real-time Quantitative Reverse Transcription PCR (Q-RTPCR)

Synthesized cDNA was measured on Q-RTPCR (SYBR Green PCR, LightCycler, Roche Diagnostics, Indianapolis, IN, USA) following the manufacturer’s recommendations. The LightCycler experimental protocol consisted of an initial Taq activation at 95°C for 8 minutes followed by a touchdown PCR step of 12 cycles consisting of 15 s at 95°C, 5 s at 68°C and 8 s at 72°C, followed by 33 cycles consisting of 15 s at 95°C, 5 s at 60°C, and 10 s at 72°C, with a single fluorescence measurement. Primers were designed with Primer3 software (Whitehead Institute/MIT, MA, USA) and purchased from Eurogentec (Seraing, Belgium). The following primers were used: Arginase 1, IL-6, inductible nitric oxide synthase (NOS2) and TNF-α. They are described in details in Table 2. All primers had T_m_s between 59°C and 61°C and all products were 100–200 bp. Cyclophilin A mRNA was used as internal standard to control amplification variation due to differences in baseline mRNA concentration. Relative mRNA levels for each tissue were computed from the threshold cycle (Ct) values obtained for the target gene, the efficiency of the primer set, and cyclophilin mRNA levels in mouse samples using RealQuant software (Roche Diagnostics, GmbH, Mannheim, Germany; version 1.01). Two independent experiments of RT-PCR were performed. Both series analyzed genes expression in M0, M1, M2 macrophages and bone marrow-derived macrophages cultured in the presence of oxygen/glucose-deprived OHCs, allowing comparison between macrophages activation programs. Only the first series additionally comprised bone marrow-derived macrophages cultured in the presence of control OHCs.

**Table 2:** Primers used in PCR reactions.

Transcript	Accession number	Sense primer	Antisense primer	Product size (bp)
*Arg1*	NM_007482	TCACCTGAGCTTTGATGTCG	TTCCCAAGAGTTGGGTTCAC	171
*Il6*	NM_031168	CTGACAATATGAATGTTGGG	TCCAAGAAACCATCTGGCTAGG	159
*Nos2*	NM_010927	AAGCTGAACTTGAGCGAGGA	TGCCCATAGGAAAAGACTG	105
*Tnf*	NM_013693	CTTGTTGCCTCCTCTTTTGC	AATGACCCGTAGGGCGATTA	151
*CypA*	NM_008907	ATGGCAAATGCTGGACCAAA	GCCTTCTTTCACCTTCCCAAA	108

*Arg1:* arginase; *Il6:* interleukin 6; *Nos: 2* nitric oxide synthase2 inducible; *Tnf:* tumor necrosis factor; *CypA:* Cyclophilin A.

### Induction of Transient Focal Cerebral Ischemia by Middle Cerebral Artery Occlusion

Adult male Sprague–Dawley rats (Centre d’Elevage René Janvier, France; 280 g body weight) were used. The intraluminal suture model was performed as previously described by Longa et al. [15]. Briefly, the rats were anesthetized and maintained by 1.0% isoflurane in air using a face mask. Rectal temperature was kept at 37°±1°C throughout the surgical procedure, using a feedback-regulated heating pad. After temporary ipsilateral common carotid artery (CCA) clamping, tMCAO was induced by advancing a filament (0.37 mm diameter, Doccol Corporation, Redlands, CA, USA) from the external carotid artery into the lumen of internal carotid artery to block the origin of the right MCA. The CCA clamp was then removed during occlusion. After 1 hour, the endovascular suture was withdrawn into the stump of the external carotid artery to allow reperfusion. For the sham group, the filament was inserted without being advanced to the MCA bifurcation. To alleviate pain, animals received 0.05 mg/kg subcutaneous buprenorphine (Temgésic, Schering-Plough Canada Inc., Kirkland, QC, Canada) immediately after induction of anesthesia.

### Behavioral Examination

Rats were familiarized with the test environment (quiet room, low light). Each session consisted in evaluation of functional recovery on 2 behavioral tests [16].

*Modified Neurological Severity Score*: The modified Neurological Severity Score (mNSS) is a composite of motor, sensory and balance tests. Neurologic function was graded on a scale of 0 to 14: the higher the score, the more severe the injury.

*Adhesive-removal somatosensory test*: Two small adhesive-backed paper dots (1 cm^2^) were used as bilateral tactile stimuli occupying the distal–radial region on the wrist of each forelimb. The rat was then returned to its home-cage. The time to remove each stimulus was recorded for 3 trials per day. Individual trials were separated by at least 5 mins. The animals had been pre-trained for 3 days before surgery.

### MR Imaging

MRI was performed on a Bruker Biospec 7T/12-cm magnet, interfaced to a Paravision 5.0 device (PV5.0, Bruker, Ettlingen, Germany), using a birdcage head-coil of 72 mm inner diameter for transmission and a 25 mm diameter surface coil for reception. The animal was placed in a non-magnetic holder equipped with a nose cone for administration of anesthetic gas (1.5% isoflurane), stereotaxic ear bars, an integrated water-heating system to maintain body temperature at 37°±1°C, and a pressure probe to monitor respiration. The MR imaging session is depicted in Figure 1B and sequences are described in Table 3.

**Table 3:** MR imaging parameters.

Imaging Parameters	Spin-echo RARE T2-weighted images		Gradient-echo FLASH T1-weightedimages	Multi-slice multi-echo (MSME) for T2 maps
TE/TR (ms/ms)		75/3,000	3.6/161.7	11.7/4,000
Flip angle (degrees)		180	90	90
No. of signals acquired		2	2	8 echoes, inter-echo delay: 23.3 ms
Bandwidth (kHz)		50	101	303
Field of view (mm^2^)		40×40	40×40	40×40
Slice thickness/Gap (mm)		1.5/0.5	1/0	1.5/0.5
Number of slices		7	15	7
Matrix size		256×256	256×256	256×92

TE = echo time; TR = repetition time.

### Contrast Agents

DOTA-gadolinium (Dotarem®, Guerbet, Aulnay-sous-Bois, France) was administered via an intravenous catheter in the caudal vein, at a dose of 0.4 mmol/kg.

P904 USPIOs (Guerbet Research, Aulnay-sous-Bois, France) were administered via the same route at a dose of 1 mmol Fe/kg. This contrast agent is composed of an 8-nm crystalline iron oxide core (maghemite γ-Fe_2_O_3_) coated with a hydrophilic material for stabilization and biocompatibility. Its mean hydrodynamic diameter is 25 nm (range: 20 to 50 nm). P904 r1 relaxivity is 1.6±0.2 mM^−1^s^−1^ at 7 T in 4% HAS (a medium close to plasma) at 37°C, and r2 relaxivity is 94±5 mM^−1^s^−1^ under the same conditions.

### MR Image Analysis

Data were processed using in-house software developed at Grenoble Institute of Neuroscience in the Matlab environment (MathWorks, Natick, MA, USA) [17]. Lesion, ipsilateral hemisphere (IH) and contralateral hemisphere (CH) were manually delineated on T2-weighted imaging. The IH:CH ratio was used to evaluate brain swelling. Infarct areas were corrected for brain swelling by dividing them by the IH:CH ratio, and infarct volumes were then calculated by integrating the resulting corrected areas over all slices, taking slice thickness into account.

T2 maps were generated by monoexponential fitting of the native data using a Levenberg–Marquardt algorithm. Because hypointense signals related to USPIO uptake were only observed in perilesional areas, 3.3 mm^2^ ROIs were systematically placed on the T2 maps in the perilesional striatum and corresponding contralateral region. Drop in T2 in the ipsilateral compared to the contralateral hemisphere was assessed as:

% T2 =  [T2(ipsilateral) - T2(contralateral)]/T2(contralateral).

### Tissue Preparation and Histology

Brains were dissected, frozen at −80°C and cut into 16 µm sections by cryostat. The sections were fixed in 4% paraformaldehyde for 15 min at room temperature. After 30 min endogenous peroxidase blocking, slices were washed with 0.1% PBS-Tween and incubated overnight at 4°C in PBS/3% BSA with monoclonal mouse anti–ED1 (AbC117-6714, 1∶1000; AbCys, Paris, France), an antibody directed against the CD68 membrane receptor present on rat macrophage lysosomal membranes. Sections were then rinsed with 0.1% PBS-Tween and incubated with biotinylated horse anti-mouse secondary antibody for 1 h at room temperature (1∶500; Vector Laboratories, Burlingame, CA, USA). Sections were then treated with extravidin-peroxidase for 1 h at room temperature (1∶1000; Sigma-Aldrich, St. Louis, MO, USA) followed by 0.2 mg/ml diaminobenzidine in 50 mmol/L pH 7.6 Tris-HCL buffer in PBS with 0.01% hydrogen peroxide until stained. Finally, sections were counterstained with nuclear red.

### Immunohistology Image Analysis

Entire brain sections were captured with a digital camera (F-View II; Soft Imaging System) using the 10× magnification microscope lens (Zeiss Axioplan II) and analyzed on ImageJ software (ImageJ 1.41, NIH, MD, USA). An ROI was first defined so as to encounter both lesional and perilesional areas. Then, the area occupied by ED1 staining was determined by automatically measuring the area of pixels above a threshold value. Finally, the area of ED1 immunostaining was indexed to the total ROI area, so as to estimate ED1 staining density. In addition, the number of ED1-immunopositive (ED1+) cells was counted manually in a 0.11 mm^2^ ROI within the perilesional area (same location as for MRI measurements).

### Statistical Analysis

Statistical analysis was performed with the SPSS 17 (SPSS Science) statistical software package for Windows. Data are given as mean ± standard deviation. Longitudinal data were assessed by analysis of variance for repeated measures using the general linear model, followed by post-hoc Bonferroni test. The number of animals in each group was based on power analysis assuming a standard deviation of 25% and a treatment effect of 30%. Animals without a complete set of data for the 2 weeks were excluded from analysis. Non-parametric Mann-Whitney tests were used for single-point comparison. Pearson correlation coefficients were calculated to assess the relationship between MRI and immunohistological endpoints. Chi*-*square tests were used to assess the difference between the proportions of animals with or without prolonged BBB disruption. Probability values of 0.05 or less were considered to indicate statistical significance.

## Results

### Macrophages Prevent Hypoxia-induced Neuron Cell Death *in vitro*

**Figure 2:**
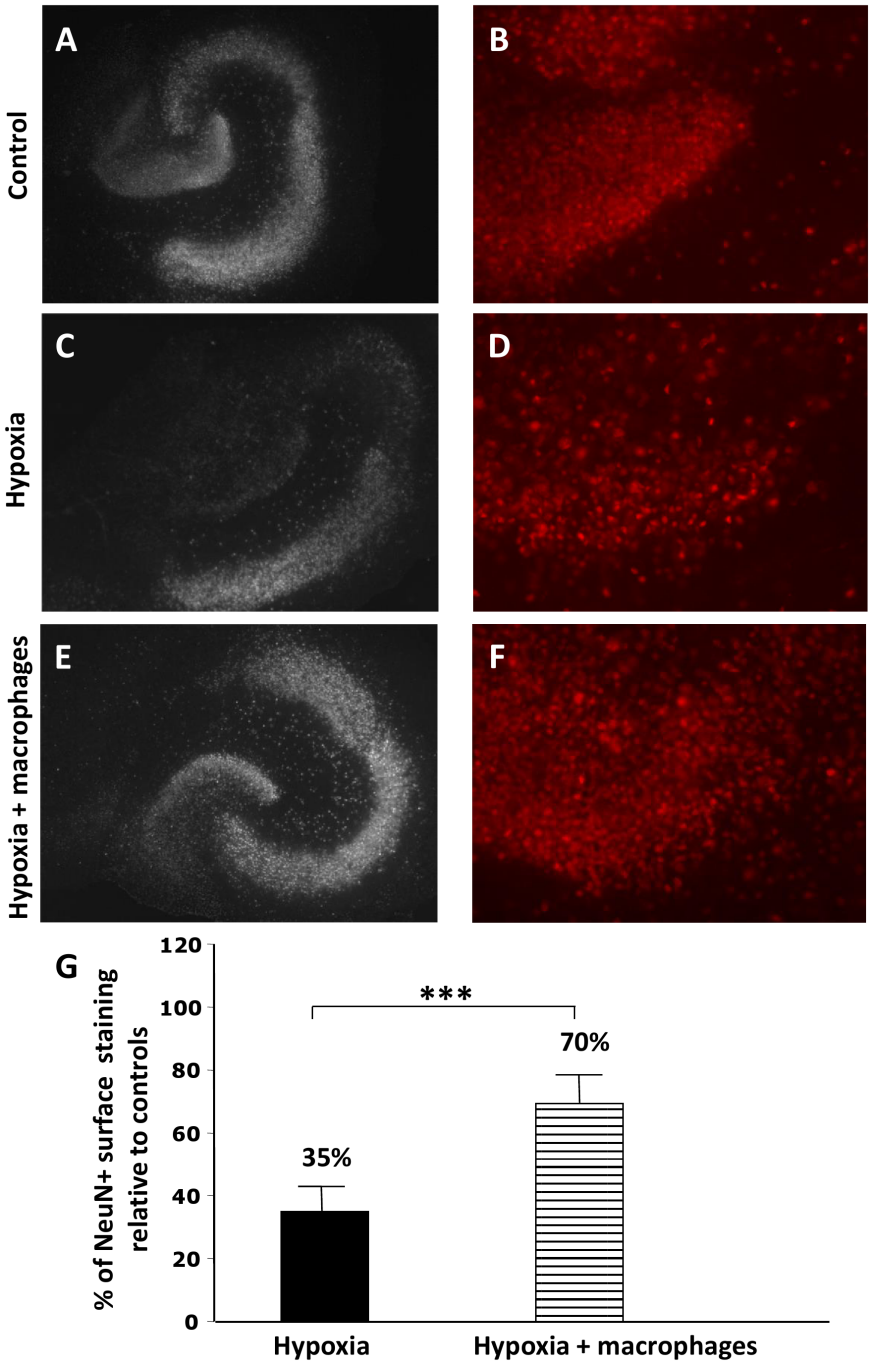
Co-cultured macrophages prevent hypoxia-induced neuronal cell death *in vitro.* **A**–**F:** NeuN staining was performed on control organotypic hippocampal cultures (control OHCs), OHCs subjected to oxygen-glucose deprivation (OGD) (hypoxia) or OHCs subjected to OGD and co-cultured with macrophages (hypoxia+macrophages). Low magnification views of hippocampal slices are shown in A, C and E. High magnification views of the dentate gyrus are shown in B, D and F. As compared to control hippocampal slices (A, B), neuron cell loss was demonstrated in oxygen/glucose-deprived OHCs (C, D) and was found to affect the dentate gyrus systematically. In contrast, oxygen/glucose-deprived OHCs that were co-cultured with macrophages appeared partially protected from OGD-induced neuron cell death. **G:** A quantitative analysis of neuron density was performed in the dentate gyrus by measuring the area covered by NeuN staining relative to the area analyzed. An arbitrary value of 100% was attributed to the measures obtained in control OHCs. Data showed a mean 35% ±8% neuron density relative to controls in oxygen/glucose-deprived OHCs (hypoxia) and that co-culture with macrophages (hypoxia+macrophages) allowed neuron density to reach 70% ±10% relative to controls (Mann-Whitney, n = 3 experiments in each group, ***: p<0.001).

In OHCs subjected to OGD, neuron cell loss could be demonstrated in different hippocampal neuron populations (dentate gyrus granular cells and pyramidal cells of Ammon’s horn), as assessed by NeuN staining (Figure 2 A–D) or DAPI coloration (data not shown). While the extent of neuron cell loss varied from one experiment to another, dentate gyrus granular neurons were systematically affected by OGD (Figure 2B, 2D). Macrophage co-culture had a striking protective effect on oxygen-glucose deprived hippocampal neurons (Figure 2E–F): compared to control OHCs, neuron survival was 35% ±8% in hypoxic slices and 70%±10% in hypoxic slices co-cultured with macrophages (Figure 2G). This represents an increase of nearly 100% in neuron survival under bone marrow-derived macrophage co-culture.

### Hippocampal Slices Subjected to Hypoxia Trigger an Alternative Activation Program in Macrophages

**Figure 3:**
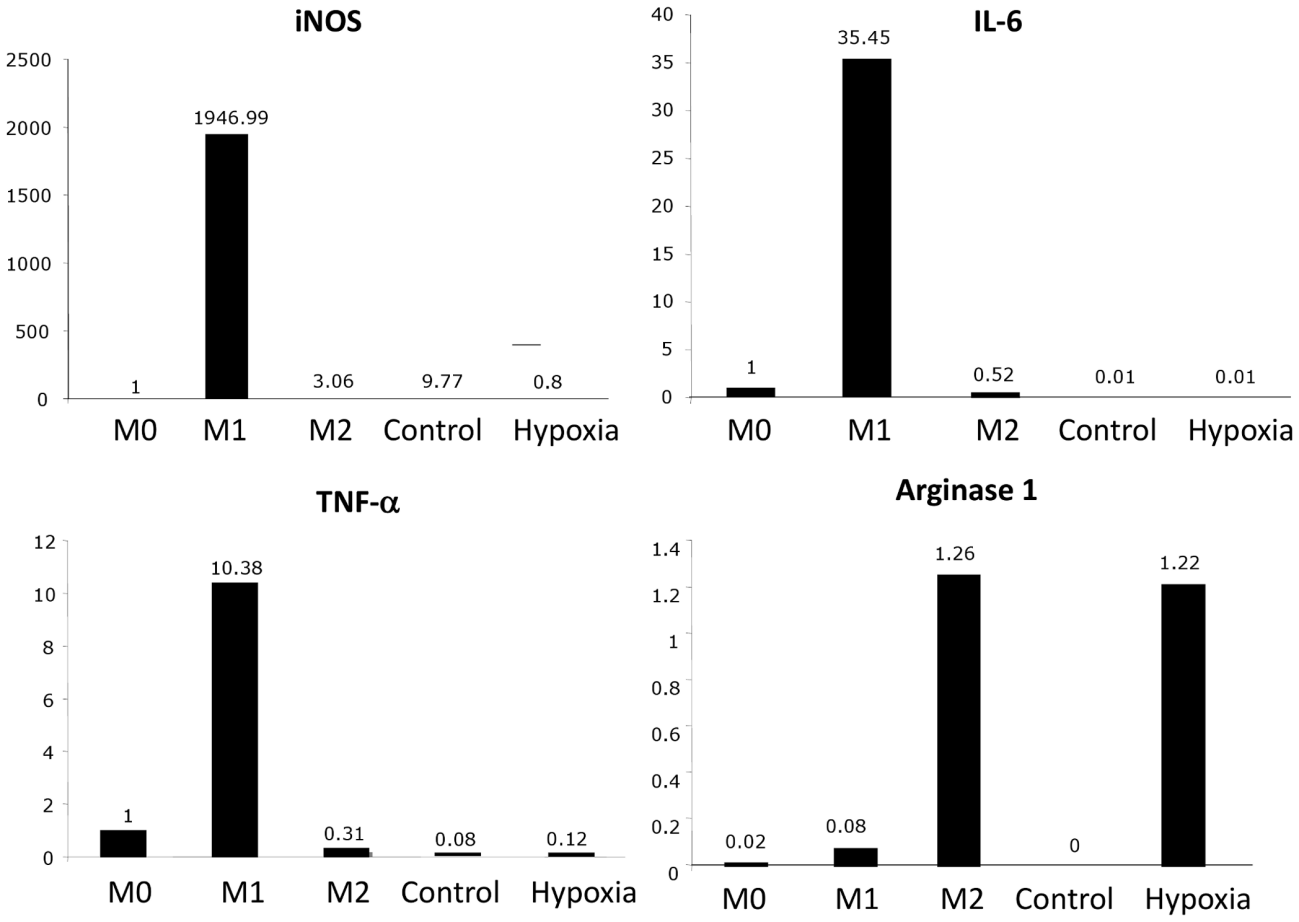
Hypoxic hippocampal slices trigger an alternatively-activated program in macrophages. Bone marrow-derived macrophages were cultured in the presence of control (Control) or oxygen/glucose-deprived OHCs (Hypoxia) in 6-well culture plates. Macrophages from each experimental condition were pooled and assessed by Q-RTPCR for the expression of the following genes: inductible nitric oxide synthase (iNOS), IL-6, TNF-α and Arginase-1. As controls, analyses were performed in parallel on mRNAs extracted from M1 macrophages (stimulated with IFN-γ), M2 macrophages (stimulated with IL-4) or M0 macrophages (non-stimulated). Data showed that, as expected, iNOS and the pro-inflammatory cytokines IL-6 and TNF-α were highly expressed in M1 macrophages as compared to M0 or M2 macrophages. With regard to these M1-type pro-inflammatory genes, bone marrow-derived macrophages co-cultured with oxygen/glucose-deprived OHCs (Hypoxia) exhibited low levels of mRNA expression and a similar profile to bone marrow-derived macrophages co-cultured with control OHCs (Control). In contrast, the M2-type gene Arginase-1 was highly expressed in hypoxic macrophages (co-cultured with oxygen/glucose-deprived OHCs) as compared to control macrophages (co-cultured with control OHCs). As expected, Arginase-1 was also highly expressed in M2 macrophages as compared to M0 or M1 macrophages. Data shown are representative of two independent experiments.

The cytokine profile of the macrophage co-cultures was then assessed at the mRNA level by Q-RTPCR examination of transcripts coding M1-type cytokines (TNF-α, IL-6, iNOS) or Arginase 1, a molecule expressed by alternatively-activated macrophages (Figure 3). In two independent experiments, macrophages co-cultured with hypoxic brain slices, did not show increased expression of M1-type genes as compared to unstimulated macrophages (referred to as M0) or M2 macrophages (obtained by IL-4 stimulation of bone-marrow derived macrophages). On the contrary, as compared to M0 or M1 macrophages, increased expression of Arginase-1 was observed in both macrophages co-cultured with OGD brain slices and M2 macrophages. Overall, these results indicate that macrophages co-cultured with OGD brain slices tend to express a M2-type alternative phenotype and did not engage a M1 activation program.

### M2 Macrophages Do Not Protect against Ischemic Damage *in vivo*

**Figure 4:**
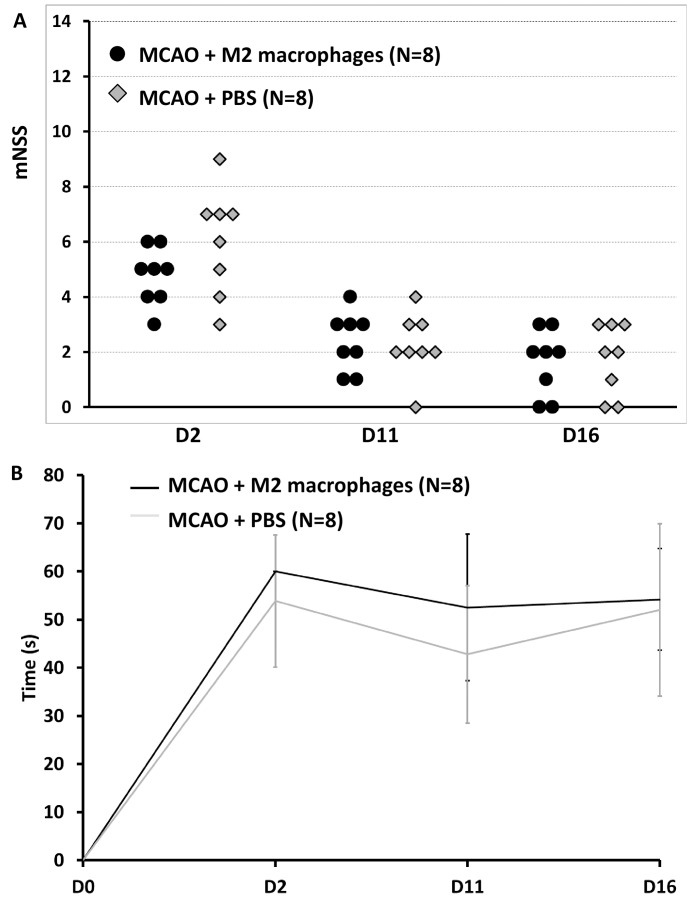
M2 macrophages do not improve functional outcome after ischemic stroke. **A-** mNSS test. **B-** Adhesive removal test. There was no statistical difference in rat performance before treatment (D2) for either behavioral test. After treatment (D11 and D16), there was an improvement in neuroscores in both treatment groups, but no statistically significant difference between them (analysis of variance for repeated measures, N = 8 in each group, p = 0.505). Similarly, time to remove the adhesive was slightly improved in both groups, without any significant difference (analysis of variance for repeated measures, N = 8 in each group, p = 0.699). PBS: phosphate-buffered saline (used as vehicle for cell delivery).

To assess the relevance of these *in vitro* findings to stroke therapy, M2 macrophages were administered in a model of focal cerebral ischemia. Table 1 summarizes the number of animals included in the study and analyzed: 8 animals were excluded at D3 because of a small, striatal-restricted, infarct; 2 animals did not have a complete set of longitudinal MRI data because of technical problems (10 animals excluded in total). Intravenous administration of 2×10^6^ M2 macrophages 4 days after reperfusion did not significantly improve functional outcome, assessed longitudinally for 2 weeks post-tMCAO with either of the behavioral tests (mNSS: p = 0.505; adhesive tape removal test: p = 0.699) (Figure 4).

**Figure 5:**
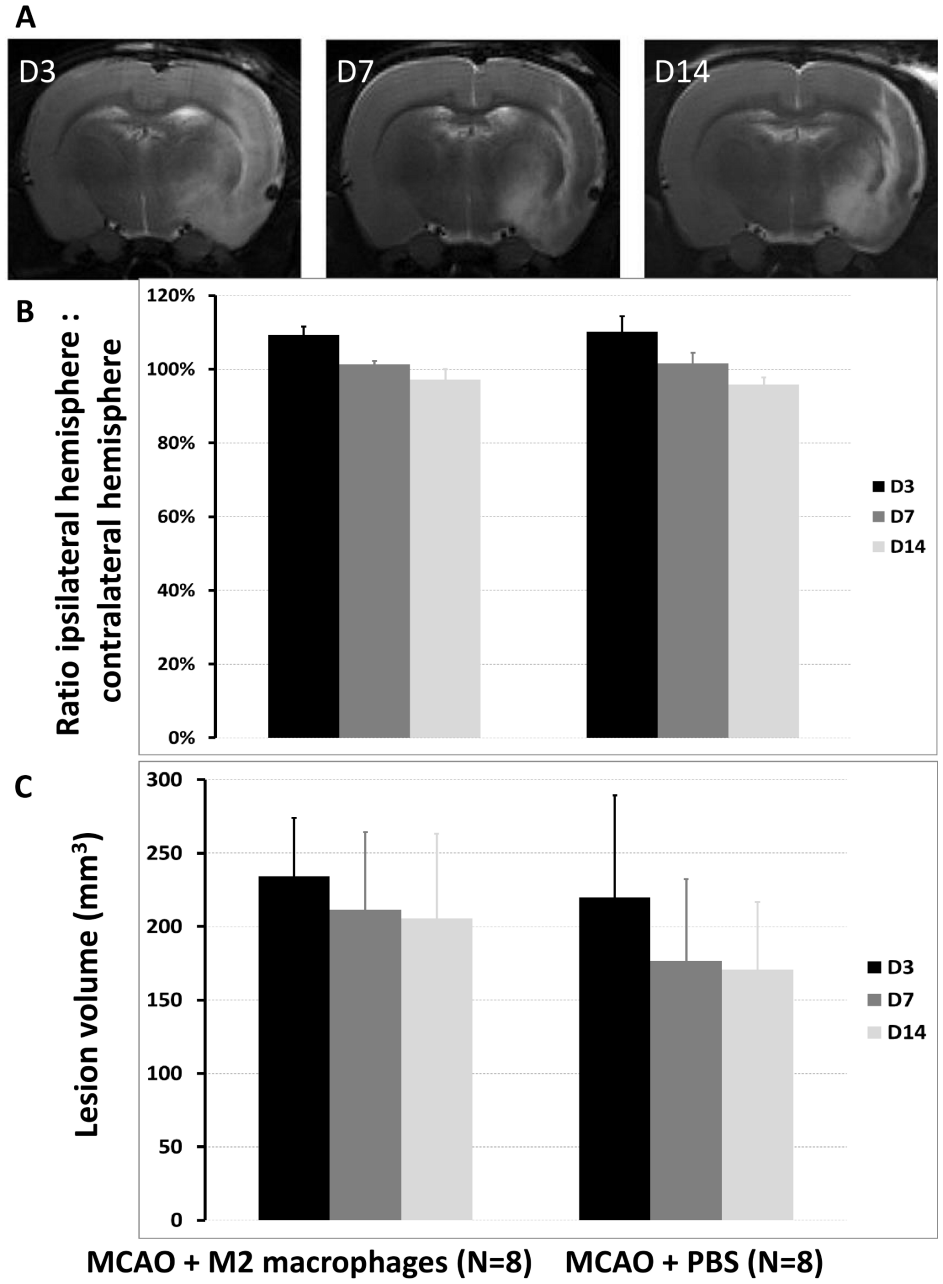
M2 macrophages do not reduce lesion size after ischemic stroke. **A-** Typical T2-weighted images obtained at D3, D7 and D14 post-ischemia showing the lesion as a hyperintense signal. **B-** The ratio of hemisphere volumes [ipsilateral:contralateral] showed moderate brain swelling at D3, normalization at D7 and moderate atrophy at D14. No statistically significant difference was seen between treatment groups (analysis of variance for repeated measures, N = 8 in each group, p = 0.941). **C-** Before treatment (D3), lesion volume (corrected for brain swelling/atrophy) was not significantly different between groups. Likewise, after treatment (D7 and D14), there was no significant difference between groups (analysis of variance for repeated measures, N = 8 in each group, p = 0.346).

Figure 5A shows the cortico-striatal lesion of 1 representative tMCAO animal at D3, D7 and D14, detected by hyperintense signal on T2-weighted images (with pseudo-normalization, the so called “fogging effect”, at D7 and D14). There was no statistically significant difference in brain swelling (p = 0.941, Figure 5B) or lesion volume (p = 0.346, Figure 5C) between treated and vehicle groups. To assess BBB integrity, T1-weighted images were acquired before and after intravenous administration of DOTA-gadolinium. Extravasation was observed in the whole lesion at D3 in all tMCAO animals. Prolonged BBB disruption, demonstrated by sustained T1 enhancement after D3, was observed in 41% (7/17) of tMCAO animals, but appeared to be independent of treatment, as the same proportion was found in the treated (3/8, 38%) and vehicle (4/9, 44%) groups (chi-squared test, p=0.7).

**Figure 6:**
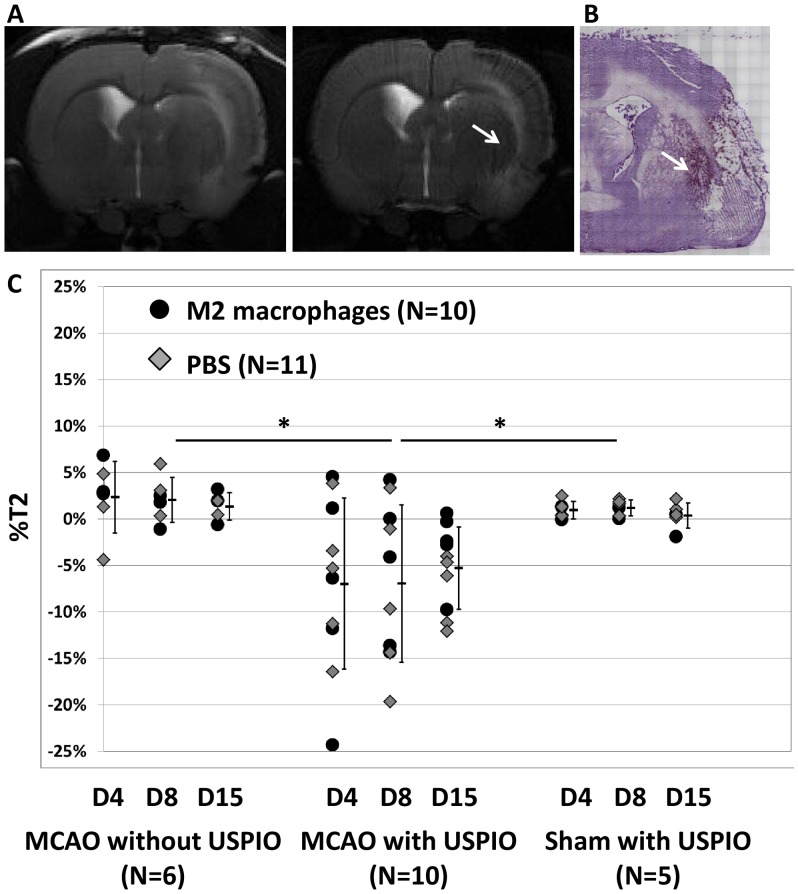
M2 macrophages do not reduce cerebral USPIO uptake after ischemic stroke. **A-** Typical T2-weighted images obtained before and 24 h after intravenous USPIO injection. The arrow shows the slight hyposignal detected in the perilesional area. **B-** Abundant ED-1+ cells were detected at the same location (arrow). **C-** Quantification of T2 difference between ipsilateral and contralateral hemisphere (%T2) showed that the T2 drop in the perilesional area was specific to tMCAO rats injected with USPIO (analysis of variance for repeated measures, *p<0.01). However, no effect of M2 treatment on T2 changes was found (analysis of variance for repeated measures, N = 5 in each group, p = 0.523).

To assess neuroinflammation, T2 maps were acquired before and 24h after intravenous USPIO administration. USPIOs are engulfed in vivo by phagocytic cells, which thus become detectable on MRI 18. A diffuse weak drop in T2 signal was seen in the perilesional area in tMCAO+USPIO animals at all time-points post-injection, regardless of the spatio-temporal pattern of BBB disruption (Figure 6A, arrow). Analyzing data quantitatively, independently of treatment group, the percentage T2 difference between ipsilateral and contralateral hemispheres was significantly smaller in the (tMCAO with USPIO) group than in the (tMCAO without USPIO) (ANOVA for repeated measures, p = 0.001) or (sham with USPIO) group (Figure 6C, p = 0.004). These results suggested that the T2 signal drop reflected iron accumulation specifically related to tMCAO, rather than a fogging phenomenon due to clearing processes [19]. This was consistent with abundant ED-1+ cells detected in the perilesional area of all tMCAO animals on immunohistology (Figure 6B, arrow). Moreover, quantitative analysis of ED1 immunostained slices revealed a significant correlation between stained area and %T2 (R^2^ = 0.803, p = 0.009). No significant effect of M2 treatment on %T2 was found in (tMCAO with USPIO) animals at any time point (ANOVA for repeated measures, p = 0.523). In line with this finding, there was no significant difference in the number of ED1+ cells between M2-treated (158±106) and control animals (186±105) (p = 0.705, Mann-Whitney test).

## Discussion

In the last decade, the concept of macrophage activation programs has shed new light on the roles exerted by the mononuclear phagocyte system under diverse pathologic conditions [13], [14], [20]. Beside the classical activation program (known as the M1 program), which can be induced by lipoplysaccharide (LPS) or interferon-γ (IFN-γ), macrophages may engage different types of alternative activation programs (known as M2 programs) that were shown to favor tissue repair, to dampen acute inflammation or, in the case of tumor-infiltrating macrophages, to support tumor outgrowth [13], [14], [20]. Signals that trigger such alternative activation programs are likely delivered in the local environment of blood-derived macrophages and notably include IL-4 and IL-10.

In the present experimental setting, hippocampal slices subjected to hypoxia released soluble factors that triggered an M2-type activation program in co-cultured bone marrow-derived macrophages. In this regard, it is noteworthy that insulin growth factor-1 (IGF-1), a cytokine known to be synthesized by M2 macrophages, was previously shown to prevent ischemia-induced neuron cell death [21], [22]. With regard to the overall neuroprotective effects of M2 macrophages, the present results are in line with previous reports that infiltrating blood-derived macrophages expressed an M2 phenotype and supported tissue repair in a model of spinal cord injury [6] and in a model of multiple sclerosis [23]. However, to our knowledge, the present study is the first to demonstrate the effectiveness of M2 macrophages in protecting neurons subjected to ischemia. This effect, mediated by soluble factors, appears similar to the neuroprotection provided by bone marrow mesenchymal stem cells (BMSCs) on oxygen/glucose-deprived OHCs [24]. This suggests that BMSCs and M2 macrophages may support survival of hypoxic neurons through common paracrine mechanisms.

Overall, the first experiment showed that M2 activated macrophages can exert neuroprotective effects under ischemic conditions. This led us to investigate the potential of using M2 macrophages in cellular therapy in focal cerebral ischemia. CBI has been emerging as a new promising modality for treating stroke since the 1990 s. Various types of cellular preparation, mainly involving stem cells, have been shown to improve functional outcome in experimental stroke [25]. To our knowledge, the present study is the first to assess M2 macrophages as CBI candidates. The results were disappointing, with no improvement in outcome, whether functional or lesional. This failure of translation from an *in vitro* to an *in vivo* model might be explained in part by delayed *in vivo* injection. In most studies reporting benefit, cells were administered during the acute phase of ischemia. Our rationale for injecting M2 macrophages during the subacute stage was twofold. Firstly, this time-window seemed more appropriate from a translational perspective, as production of M2 macrophages requires time. Secondly, this time-window corresponded to a plateau for the recruitment of blood-derived macrophages [26]. This led us to assume that the microenvironment (cytokines, chemokines and adhesion molecules) would be optimal for macrophage migration into the brain parenchyma. However, bone marrow-derived M2 activated macrophages might lack receptors to enter the brain, such as the chemokine receptor CCR2 [27]. This could explain in part the lack of benefit, as the CCR2-dependent recruitment of inflammatory monocytes and their subsequent differentiation within the lesioned brain environment into noninflammatory phagocytes has been recently shown to be a key mechanism linking postischemic brain inflammation with repair [3].

On the other hand, immunomodulatory cells may not need to enter the brain to elicit an effect, but may act in the periphery to increase trophic factor expression in the brain [28]. This idea is supported by the fact that the neuroprotective effect observed in co-cultures of oxygen/glucose deprived OHCs with macrophages was obviously mediated by secretion of protective soluble factors without a need for direct cell-to-cell contact. The present results may thus also be explained in part by dilution and/or insufficient release of these factors after intravenous injection – a delivery route chosen as being minimally invasive. Alternatively, endovascular drug delivery could be performed by interventional physicians and is potentially more comfortable for patients than an intracranial route. However, a potential danger of this approach is that cells might aggregate during the process of injection, forming microemboli. In fact, our team has previously shown that intra-carotid infusion of bone marrow-derived macrophages increased mortality and induced brain lesions in a tMCAO model [29].

### Limitations

The issues remaining to be solved before moving on to clinical applications of cell therapy in ischemic stroke have been recently discussed and constitute the road map for the STEPS consortium [11]. The present study had several limitations in the light of these recommendations. Firstly, additional *in vitro* studies would be needed to identify macrophage-derived factors that support neuronal protection and neuronally-derived molecules that trigger macrophage activation. It would also be of interest to determine whether or not M1 activated macrophages afford a similar protection to hypoxic neurons. Secondly, we did not perform any *in vivo* dose-response study. The *in vivo* cell dose tested was, however, based on a study by Mikita et al. [23], reporting therapeutic benefit with M2 activated macrophages in a model of multiple sclerosis, and was consistent with the cell dose chosen in most studies of cellular therapy using intravascular administration. Of note, however, in the study performed by Mikita and coll [23], M2 macrophages were administered twice with a delay of 2 days to induce a strong clinical and histological benefit. The experimental design of the current study would allow such a second M2 administration at D6. Additional experiments are thus needed to completely explore the therapeutic potential of M2 macrophages beyond the single dose trial. Also, other ways of administration (intra-cerebral, intra-arterial) might prove more efficient despite their invasiveness. Thirdly, animal follow-up was limited to 2 weeks, whereas longer term follow-up (up to one month) is usually recommended. Nonetheless, the fact that no endpoint differed between groups during this 2-week time-span does not offer much hope for any subsequent improvement. Finally, we did not study cell trafficking and final fate following i.v. injection. Many macrophages might get trapped in other organs such as the lungs and the liver. A non-invasive modality such as MRI could monitor the delivery and tracking of exogenously administered cells; however, as mentioned above, cells were not especially expected to migrate into the brain, and the sensitivity of MRI might be too low to detect small amounts of cells in the brain parenchyma.

### Conclusion

In conclusion, the present study failed to demonstrate a protective effect of M2 macrophages administered intravenously in the subacute stage of transient focal cerebral ischemia, despite their neuroprotective effects observed *in vitro*. Immunomodulatory therapy, such as M2 macrophage supplementation in peripheral blood, has to take into account the complexity and dynamics of both post-ischemic cerebral inflammation and post-ischemic blood-brain barrier alterations.
